# Bactericidal activity of PA-824 against *Mycobacterium tuberculosis* under anaerobic conditions and computational analysis of its novel analogues against mutant Ddn receptor

**DOI:** 10.1186/1471-2180-13-218

**Published:** 2013-10-01

**Authors:** Sulochana Somasundaram, Ramaian Santhaseela Anand, Perumal Venkatesan, Chinnambedu N Paramasivan

**Affiliations:** 1 Department of Biotechnology, Sri Venkateswara College of Engineering, Sriperumbudur, India; 2 Centre for Biotechnology, Anna University, Chennai, India; 3 National Institute for Research in Tuberculosis, Chennai, India; 4 Foundation for Innovative New Diagnostics, Flat No. 6-14 (excluding No. 7), 9th floor, Vijaya Building, 17-Barakhamba Road, New Delhi 110 001, India

**Keywords:** Bactericidal activity, *Mycobacterium tuberculosis*, Anaerobic activity, Docking, Ddn, PA-824

## Abstract

**Background:**

The resurgence of multi-drug resistant tuberculosis (MDR-TB) and HIV associated tuberculosis (TB) are of serious global concern. To contain this situation, new anti-tuberculosis drugs and reduced treatment regimens are imperative. Recently, a nitroimidazole, PA-824, has been shown to be active against both replicating and non-replicating bacteria. It is activated by the enzyme Deazaflavin-dependent nitroreductase (Ddn) present in *Mycobacterium tuberculosis* which catalyzes the reduction of PA-824, resulting in the release of lethal reactive nitrogen species (RNS) within the bacteria. In this context, PA-824 was analyzed for its activity against latent tuberculosis under anaerobic conditions and compared with rifampicin (RIF) and pyrazinamide (PZA). Recent mutagenesis studies have identified A76E mutation which affects the above mentioned catalysis and leads to PA-824 resistance. Hence, novel analogues which could cope up with their binding to mutant Ddn receptor were also identified through this study.

**Results:**

PA-824 at an optimum concentration of 12.5 μg/ml showed enhanced bactericidal activity, resulting in 0 CFU/ml growth when compared to RIF and PZA at normal pH and anaerobic condition. Further docking studies revealed that a combinatorial structure of PA-824 conjugated with moxifloxacin (ligand 8) has the highest binding affinity with the wild type and mutant Ddn receptor.

**Conclusions:**

PA-824 has been demonstrated to have better activity under anaerobic condition at 12.5 μg/ml, indicating an optimized dose that is required for overcoming the detoxifying mechanisms of *M*. *tuberculosis* and inducing its death. Further, the development of resistance through A76E mutation could be overcome through the *in silico* evolved ligand 8.

## Background

It is estimated that one-third of the world’s population is infected with *M*. *tuberculosis* and 8.7 million suffer from active TB and 1.4 million deaths occur due to it every year [1]. *M*. *tuberculosis* is able to evade the human immune response in part by triggering formation of insulating hypoxic granulomas following infection of pulmonary macrophages. Bacilli within this environment have adapted themselves to slowly replicate and respire, making them tolerant of many drugs. This resistant state is thought to contribute to the prolonged combination chemotherapy required to cure patients [2,3]. Lack of compliance with treatments lasting up to 9 months contributes to the emergence of resistant strains [4]. To contain this situation, new anti-tuberculosis drugs and lesser duration of treatment are of immediate requirement. The discovery of new drugs involves several constraints that discourage many companies from investing in novel anti-TB drugs. The research is expensive, slow and difficult, and it requires specialized facilities for handling *M*. *tuberculosis*. Application of bioinformatics in designing novel drugs to tackle intractable TB has become feasible [5]. PA-824 is a nitroimidazole, a class of novel anti-bacterial agents. As a potential TB therapy, it has many attractive characteristics including, its novel mechanism of action, its *in vitro* activity against all tested drug-resistant clinical isolates, and its activity both as a potent bactericidal and sterilizing agent in mice. In addition, the compound shows no evidence of mutagenicity in a standard battery of genotoxicity studies, no significant cytochrome P450 interactions, and no significant activity against a broad range of Gram-positive and Gram-negative bacteria [6]. Murine model and a pre clinical study showed a substantial activity on persisters [7,8]. These reasons necessitate us to characterize the *in vitro* activity of PA-824 under anaerobic conditions, a home for persisters. Further, an *in silico* derivative of PA-824 is proposed that could act under a key resistance mutation (A76E), attributed to cause PA-824 resistance in *M*. *tuberculosis*[9].

## Methods

### Drugs

PA-824 was provided by the Global Alliance for Tuberculosis Drug Development through Doris Rouse of Research Triangle Institute (Research Triangle Park, NC). PA-824 was prepared in Dimethyl Sulfoxide (DMSO); Pyrazinamide (PZA) (Sigma) in sterile distilled water and Rifampicin (RIF) (Sigma) in dimethyl formamide (DMF). They were sterilized by filtration through cellulose membranes with a pore size of 0.22 μm, and further dilutions were then made in sterile distilled water.

### *In vitro* oxygen depletion assay for* M*. *tuberculosis*

The protocol used for the *M*. *tuberculosis* - *in vitro* oxygen depletion assay was a slight modification of the method described by Wayne and Hayes [10] and Wayne’s Nonreplicating Persistence-2 (NRP-2) model [11]. Briefly, mid-log-phase aerobic *M*. *tuberculosis* H37Rv cultures were prepared in 10 ml of 7H9 liquid medium with Tween 80-albumin-dextrose by inoculating *M*. *tuberculosis* H37Rv and incubating at 37°C for 5–7 days and the number of organisms were counted by using Thoma counter (Neubauer). Known volume (10^6^ organisms/ml) was inoculated into 18.6 ml of Dubos medium at a normal pH in 28 ml screw-cap McCartney bottles (universal containers) from Fishers Scientific Co Ltd., with methylene blue dye (1.5 g/ml) as an indicator of oxygen depletion. The blue dye fades and finally disappears under anaerobic conditions, as described by Wayne and Hayes [10]. Two to three mm diameter hole was made on the lid with rubber septa, of the containers and the mouth was sealed with parafilm. The H37Rv culture was grown at 37°C in an orbital shaker (Cetromat) at 1000 rotations /min with slow stirring for 21 days. It was shaken steadily but not very actively, to keep the bacilli in suspension and to prevent from clumping. The culture was grown under closed caps with a limited headspace. After 21 days, O_2_ in the headspace was consumed and the bacteria were in a non-replicating and low metabolic state called NRP 2 stage. Then 0 day (Viable count) VC was set up on 7H11 agar plates and the drugs were added at different concentrations.

### Bactericidal action of the drugs

PA-824 was injected at two different concentrations of 3 μg/ml (P1), 12.5 μg/ml (P2), and RIF & PZA were injected at 1 μg/ml and 50 μg/ml respectively through the septa of 21-day-old cultures. Culture bottles were prepared in duplicates for each concentration of the drugs. The culture was removed by means of a syringe through the septa and the VC was set up on 2nd, 4th, 7th, 10th, 14th, and 21st days. The cultures were serially diluted in saline and plated onto 7H11/OADC agar (Difco) plates in duplicates containing polymyxin B (200 U/ml), amphotericin B (20 μg/ml), carbenicillin (100 μg/ml), and trimethoprim (10 μg/ml), to determine colony-forming unit (CFU) counts. The plates were placed in polythene bags, along with a plate inoculated with *Mycobacterium phlei* and incubated at 37°C. *M*. *tuberculosis* colonies were counted at 0, 2, 4, 7, 11, 14 and 21 days of incubation. The results were represented, as the mean of the quadruplicates of the cultures for every time point for every drug concentration and for the control cultures it was the mean of duplicates (Table 1).

**Table 1:** **Bacterial count in Log**_**10 **_**cfu**/**ml with standard deviation on different days**

**Days**	**0**	**2**	**4**	**7**	**11**	**14**	**21**
No drug	6.55 ± 0.16	6.68 ± 0.23	6.58 ± 0.13	6.28 ± 0.23	6.35 ± 0.12	6.37 ± 0.09	6.53 ± 0.07
P1 (3 μg/ml)	6.64 ± 0.39	6.45 ± 0.08	6.48 ± 0.22	6.21 ± 0.19	6.20 ± 0.17	5.62 ± 0.54	4.93 ± 0.32
P2 (12.5 μg/ml)	6.67 ± 0.25	5.44 ± 0.44	4.69 ± 0.12	4.18 ± 0.41	4.18 ± 0.51	4.15 ± 0.09	0
RIF (1 μg/ml)	6.93 ± 0.04	6.54 ± 0.13	6.62 ± 0.05	5.2 ± 0.28	5.35 ± 0.06	4.60 ± 0.4	4.59 ± 0.48
PZA (50 μg/ml)	6.08 ± 0.39	6.84 ± 0.02	6.83 ± 0.03	6.30 ± 0.13	6.02 ± 0.44	6.33 ± 0.3	6.49 ± 0.06

### Statistics

The results were expressed as the mean of the duplicates at each time point. Differences in the regression coefficients of the log CFU counts with different drug combinations were tested by analysis of variance using test command in Stata, release 8 (Stata Corp, College station Tx). The standard deviation (SD) of a result was obtained from the variation between CFU counts on the duplicate cultures, estimated separately for the log phase and the stationary phase cultures.

### Graphing

No adequate representation on a logarithmic axis of the CFU count could be made of counts that yielded no colonies since log 0 is minus infinity. A line was therefore drawn to extrapolate the values obtained at the two previous time points provided that it cut the X axis to the left of the time point yielding no colonies. Otherwise, the line was drawn through Log 0. In each case, the line concerned has been drawn dotted to indicate the uncertainty in its true position. Counts after the first negative count always failed to yield colonies, and their values have not been entered in the graphs.

### Docking tools

The binding affinity of the analogues were obtained using AUTODOCK Vina tool with AMBER force field and Monte Carlo simulated annealing [12]. The dockings were performed in a 64 bit PC. The receptor design was made by using SWISS-MODEL, a fully automated protein structure homology-modeling server. In this tool, energy minimization and simulated annealing are done with the GROMOS96 forcefield [13]. The 2D structures of the ligands were drawn, optimized with full hydrogen bonds and saved as .sk2 format using ChemSketch tool from Advanced Chemistry Development, Inc. (ACD/ChemSketch, [14]) and the 3D structures were obtained using PRODRG server [15].

### Receptors

The wild type receptor was derived from the crystal structure of deazaflavin dependent nitroreductase (3R5W) [16]. The mutant receptor was designed by introducing A76E mutation [9], in the sequence of Ddn and modeling it using SWISS MODEL without the presence of its cofactor F420.

### Ligands

The ligands were derived from the structure of PA-824 by removing the trifluoromethyl group (CF3) and replacing it with key anti-*M*. *tuberculosis* drugs such as isoniazid, moxifloxacin, gatifloxacin etc., through ester linkages. The removal of trifluoromethyl group was done on the basis to reduce the toxicity [17]. The designed combinatorial ligands are listed in Table 2.

**Table 2:** **Docking values of PA**-**824 and its novel analogues with the wild and mutant Ddn receptor**

**S.no.**	**Drug**	**Docking score with wild type receptor (kcal/mol)**	**Docking score with A76E mutant receptor (kcal/mol)**	**Structure of the analogues**
**1**	PA-824	−6.9	−6.7	
**2**	Ligand 1 (glucose)	−7.6	−7.0	
**3**	Ligand 2 (Nitroglucose)	−7.6	−7.2	
**4**	Ligand 3 (Hydroxyl modification)	−6.3	−6.3	
**5**	Ligand 4 (Biotin)	−7.1	−6.7	
**6**	Ligand 5 (Cholestryl ester)	−8.3	−6.9	
**7**	Ligand 6 (gati)	−8.4	−8.1	
**8**	Ligand 7 (isoniazid)	−7.8	−7.5	
**9**	Ligand 8 (Moxi)	−7.7	−8.5	
**10**	Ligand 9 (PZA)	−7.2	−7.2	
**11**	Ligand 10 (Trehalose)	−8.0	−7.7	

### Analysis of binding

The binding sites for the docking were designed such that the entire receptor molecule was included within the selection grid. The highest binding energy values corresponding to the RMSD value of zero were considered as the binding affinity value of the ligands for each docking. The Hydrogen bond interactions were obtained using Molegro molecular viewer (molegro.com) [18].

## Results

### Bactericidal activity

**Figure 1:**
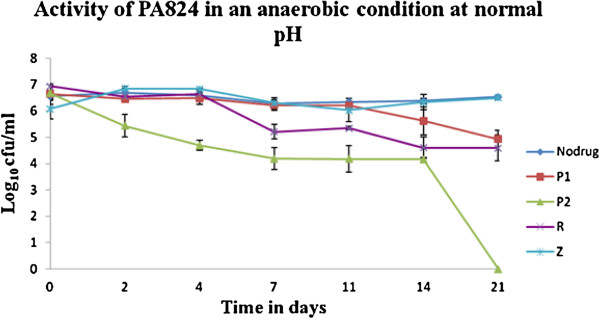
**Bactericidal activity of PA-824 on *Mycobacterium tuberculosis *H37 RV under anaerobic condition.** The treatment with 12.5 μg/ml of PA-824 shows a complete reduction in the log CFU/ml after 21 days. P1 and P2: PA-824 at 3 and 12.5 μg/ml; R: Rifampicin at 1 μg/ml; Z: Pyrazinamide at 50 μg/ml.

The results of the bactericidal activity of different drugs from the two sets of experiments are given in Figure 1. PA-824 at lower concentration of 3 μg/ml (P1) had less activity on all the days resulting with a log of 4.9 CFU/ml on the 21st day. Rifampicin (1 μg/ml) showed slightly increased activity than PA-824 at a lower concentration of 3 μg/ml, with a reduction in the count of 1.42 log cfu/ml on the 7th day, whereas for PA-824 at a concentration of 12.5 μg/ml (P2), showed a decrease in the count to log of 2.49 CFU/ml on the same day. A small reduction in RIF activity was seen on the 7th day, and on 14th day reduction of 2.34 CFU/ml was observed and with no substantial change thereafter, whereas the activity of PA-824 at a concentration of 12.5 μg/ml continued to show a steady activity and resulted in a 0 CFU/ml on the 21st day. Since the experimentation was performed in non-acid condition, the activity of PZA was not efficient without any change in the log CFU/ml up to 21st day. Since PZA is not active in normal pH medium as it needs acidic environment for its action, our findings of low PZA activity in non-acidic pH fit with this established fact (Table 1).

### Docking studies

The docking studies (Table 2) showed that Ligands 6 and 10 have the highest binding affinity of −8.4 and −8.0 Kcal/mol respectively with the wild type Ddn receptor when compared to that of PA-824 which had a value of −6.9 Kcal/mol. Considering the mutant receptor, the binding of PA-824 was lowered to a value of −6.7 Kcal/mol showing that the active site mutation has a potential to lower the binding affinity. This trend was also followed in Ligands 6 and 10 whose binding affinity values were lowered to −8.1 and −7.7 Kcal/mol respectively. Ligand 8, contradicted this trend showing an increase from −7.7 Kcal/mol with the wild type receptor to a value of −8.5 Kcal/mol with the mutant receptor. Considering that ligand 8 has a higher affinity to the wild type receptor itself than the PA-824, future evaluations of this lead could be effected.

## Discussion

### Bactericidal activity

The main aim of people, who are working for the control of tuberculosis, is to have a shorter treatment regimen than shorten the current six months duration. Following fluoroquinolones, few promising drugs were developed including nitroimidazo-oxazine PA-824, developed by Global Alliance for tuberculosis and which is in Phase II studies [7]. It has been shown that PA-824 has a novel mechanism of action affecting protein and lipid synthesis of *M*. *tuberculosis* and has potential bactericidal activity, which is comparable to that of isoniazid, a first line Anti-tuberculosis drug [8]. PA-824 also appears to be active against non-replicating bacilli, which suggests that it might be a potent sterilizing drug [19]. Hence the *in vitro* study was undertaken with PA-824, to understand its bactericidal activity on static and anaerobic *M*. *tuberculosis*. After adaptation to micro aerophilic culture, the organisms do not multiply and the drugs that are capable of killing non-replicating bacteria are useful in treating latent infection with TB. This helps to determine the sterilizing activity on *M*. *tuberculosis* in our experiments with single drugs This study observed that the activity of PA-824 at the higher concentration of 12.5 μg/ml, was greater than standard drugs such as RIF and PZA, which are known to have significant sterilizing activity (Table 1 and Figure 1).

Lenaerts *et al*., [20] showed that with 10ug/ml, PA-824 treatment under anaerobic conditions a reduction of 0.99 CFU/ml, from 6.42 to 5.43 CFU/ml was observed at the end of 28 days (24 days of anaerobic culture + 4 days of drug treatment), compared to 6.42 CFU/ml in the control. In our study, treatment with 12.5 μg/ml of PA-824 showed a reduction to 4.69 ± 0.12 CFU/ml from 6.58 ± 0.13 CFU/ml after 4 days of treatment, a net reduction of 1.89 CFU/ml which is higher than the reduction observed by Lenaerts *et al*., with 10 μg/ml. Further, treatment with 2 μg/ml of PA-824 Lenaerts *et al*., [20] showed a reduction of 0.81 CFU/ml from 6.42 to 5.61 CFU/ml compared to control. In this study with 3 μg/ml of PA-824, a similar reduction of persisting *M*. *tuberculosis* count from 6.53 ± 0.07 to 4.93 ± 0.32 CFU/ml (a reduction of 1.6 CFU/ml) in 21 days was observed. This shows an approximate doubling of the killing activity (0.81 to 1.6 CFU/ml) when the concentration and time are varied from 2 μg/ml (4 days) to 3 μg/ml (21 days). An increase in the treatment concentration to 50 μg/ml of PA-824 for 4 days in the study by Lenaerts *et al*., resulted in reductions to 5.24 CFU/ml whereas the treatment of 12.5 μg/ml of PA-824 for 21 days, which is a long term duration, resulted in complete reduction in the *M*. *tuberculosis* viable count. This could signify an important role of concentration and duration of PA-824 treatment that is required to control the persisting *M*. *tuberculosis*.

Considering the role of PA-824 as a NO donor, excess production of NO in the intracellular environment could fuel the growth of *M*. *tuberculosis* through its ‘truncated hemoglobin’ N (trHbN) detoxification machinery. In *M*. *tuberculosis* H37Ra, the activity of the *glbN* gene encoding trHbN is upregulated by the general nitrosative stress inducer, nitrite, by the NO releaser sodium nitroprusside and by hypoxia. The activity of the *glbN* gene is also enhanced during *M*. *tuberculosis* H37Ra invasion of THP-1 activated macrophages (producing NO) [21]. In *in vivo*, the high oxygen affinity of trHbN (P_50_ ~ 0.01 mm Hg) may ensure a low but critical level of oxygen, granting survival of *M*. *tuberculosis* in the granuloma hypoxic environment when the bacilli enter latency [22]. It has been proposed that the oxygenated trHbN (oxy-trHbN) catalyzes the rapid oxidation of nitric oxide to innocuous nitrate with a second-order rate constant (k’_NOD_ ≈ 745 × 10^6^ m^-1^ · s^-1^), which is 15 and 34 fold faster than the reaction of horse heart and sperm whale myoglobin, respectively [23,24]. The resulting nitrate, the most effective alternate terminal electron acceptor after molecular oxygen, could protect the *M*. *tuberculosis* from hypoxic, acid and RNS stress [25].

From crystallographic studies, it is proposed that residue Phe^62^ of trHbN exists in two conformations. In one, the benzene side chain of the residue blocks the longer channel of the tunnel path (the so-called closed state) and in the other it does not (the open state) [26,27]. By long Molecular dynamics (MD) simulations (0.1 ms), Bidon-Chanal *et al.* have proposed that in deoxytrHbN, the Phe^62^ adopts the closed conformation and hence the O2 ligand enters the protein via the short channel. In case of oxygenated trHbN, the Phe^62^ prefers the open conformation, thus facilitating the entrance of the second ligand (NO) via the long channel [28,29]. MD simulations [30] have revealed two additional tunnels: EH (EHT) and BE (BET). The conformational change from an open state to a closed state is more rare than the opposite, indicating the presence of a larger energy barrier for an open-to-closed transition. For the oxy-trHbN, the open state conformer is found 1.5 kcal/mol more stable than the closed conformer. The energy barrier for closed to open transition is ~1.2 kcal/mol whereas the reverse energy barrier is >3 kcal/mol [31]. Adding to this, trHbN matrix can hold more than one NO molecule at the same time. Further •NO diffuses from the bulk solvent through the channel to an internal cavity (EHc) of the trHbN molecule. This cavity is located between the tunnel (EHT) entrance and the side chain of the Phe^62^ residue. To reach EHc from the bulk, a NO must cross a bottleneck region of 1.3 Å radius at the protein surface [30]. This could be favored by the presence of diffusion pressure under high NO concentrations generated by treatment with excess PA-824. Further excess production of NO in the intracellular environment could regulate autophagy, which is a host derived mechanism for the endocytosis of *M*. *tuberculosis* and killing it by fusion with lysosome [32,33]. Thus excess generation of NO itself could hinder the effectiveness of killing the bacteria.

**Figure 2:**
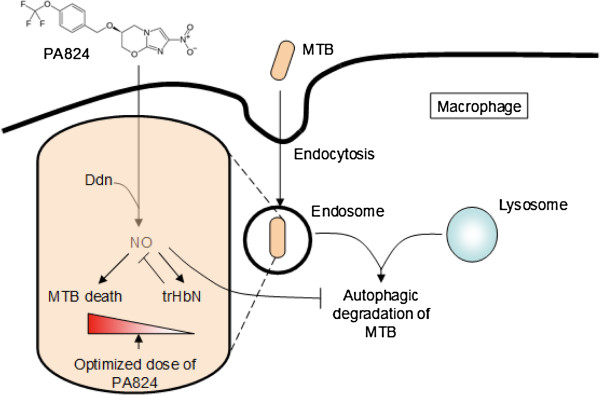
***M. tuberculosis *pathways associated with the dosage optimization for PA-824 treatment.** Excess NO release during elevated PA-824 concentrations could favor *M*. *tuberculosis* antioxidant defense mechanism involving trHbN and down regulation of autophagy. Hence dose optimization of PA-824 therapy is a key parameter for successful killing of the pathogen.

This triggering of the detoxification machinery by NO highlights the importance of dose and treatment duration optimization in PA-824 therapy which could otherwise fuel the antioxidant survival strategies of *M*. *tuberculosis* outlined in the above discussion (Figure 2). This is also evident from the phase II clinical studies wherein increasing the PA-824 doses resulted in an unchanged Early bactericidal activity (EBA), with a steady decrease in the number of TB bacteria in the sputum (~0.1 log drop in CFU per day for 14 days, as compared with 0.148 for the standard regimen). This means that maximum effectiveness was seen at the lowest dose tested: 200 mg [7]. The 12.5 μg/ml concentration of PA-824 and 21 days of treatment observed in this study could enhance the clearance of *M*. *tuberculosis* by overcoming its detoxification machinery. Thus the optimum dosage and treatment duration could provide better insights in setting the clinical evaluations using free drug concentration greater than MIC (T_>MIC_) as a parameter [34].

With respect to RIF, the findings of our study are similar to that of an *in vivo* model in mice, showing that PA-824 was more active than RIF with more activity on the metabolically active organisms but not on non-replicating organisms [20]. Since the culture is in a pH of 6.8, as expected the PZA activity was constrained which has no bactericidal activity in non-acidic environments and the growth line in the graph (Figure 1) is similar to that of no drug. PZA had more sterilizing activity on slow multiplying organisms in an acidic condition inside macrophages [35], whereas PA-824 had more sterilizing activity on non-replicating persisters.

### Docking studies

**Figure 3:**
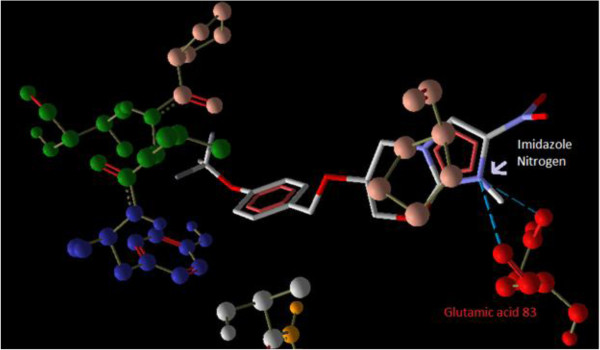
**Interaction of PA-824 with the active site of wild type receptor show two hydrogen bond interaction (blue dotted lines) of the imidazole nitrogen (Position 7) with the two hydroxyl oxygens of glutamic acid 83 (red) of the Ddn receptor.**

**Figure 4:**
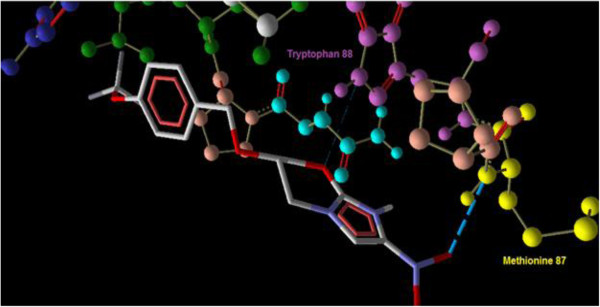
**Interaction of PA-824 with the active site of mutant receptor shows the two hydrogen bonds (blue dotted lines).** The oxygen of Nitro group interacts with Methionine 87 while the oxygen atom at position 8 interacts with Tryptophan 88.

**Figure 5:**
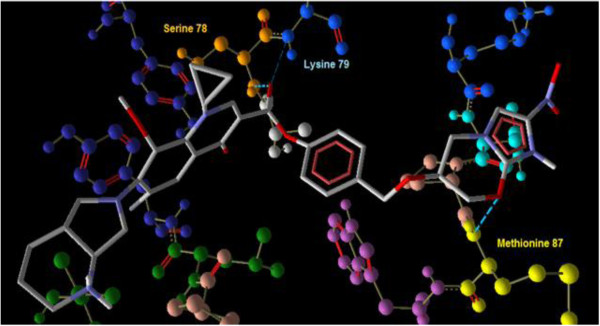
**Interaction of ligand 8 (Moxi) with the active site of mutant receptor shows three hydrogen bond interactions (blue dotted lines).** The carbonyl oxygen shows interaction with Serine 78 (orange) and Lysine 79 (blue) and the oxazine oxygen shows interaction with Methionine 87 (yellow).

**Figure 6:**
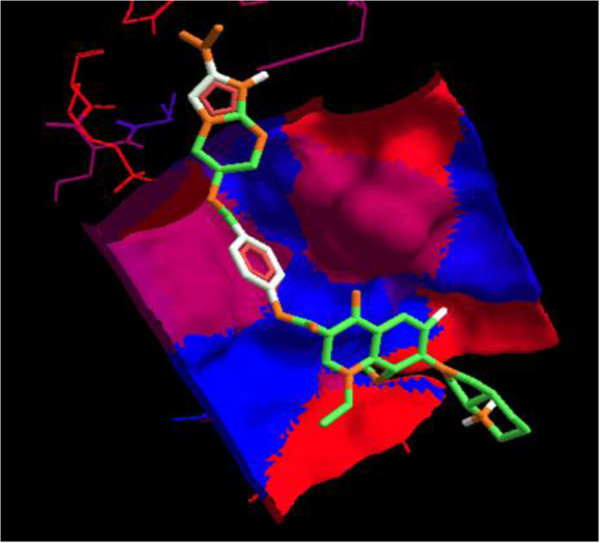
**Interaction of ligand 8 with the wild type receptor showed no key hydrogen bond interactions.** While the hydrophobicity of the binding pocket could contribute to the high binding affinity value of −7.7 kcal/mol.

**Figure 7:**
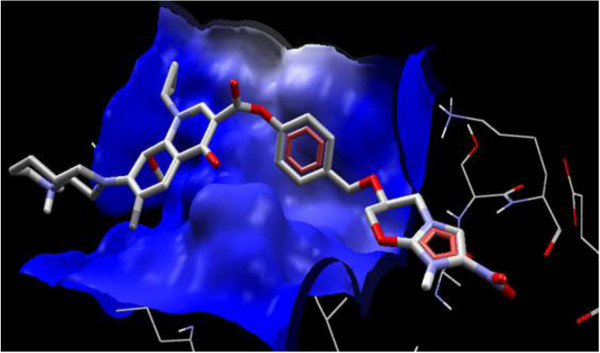
**Ligand 8 electrostatic interaction with the active site of wild type receptor shows a favourable plateau of electrostatic interaction which could account for its binding affinity value of −7.7 kcal/mol.**

Interaction of PA-824 with the active site of wild type receptor show two hydrogen bond interaction of the imidazole nitrogen (Position 7) with the two hydroxyl groups of glutamic acid 83 represented in red (Figure 3). Interaction of PA-824 with the active site of mutant receptor shows a total of two hydrogen bonds. The oxygen of Nitro group interacts with Methionine 87 while the oxygen atom at position 8 interacts with Tryptophan 88 (Figure 4). These interactions show that the key hydrogen bonding with Glutamic acid 83 present in the wild type receptor is absent in the mutant receptor. Ligand 8, which showed a high affinity with the mutant receptor showed a different scenario of binding with three hydrogen bond interactions (Figure 5). The carbonyl oxygen showed interaction with Serine 78 (orange) and Lysine 79 (blue) and the oxazine oxygen showed interaction with Methionine 87 (yellow). The Serine 78 residue in the Ddn receptor is essential for the binding of F420, a cofactor involved in Ddn activity, and PA-824 [16]. Thus further investigation of the PA-824 binding in the presence of F420 cofactor needs to be evaluated. Interestingly, interaction of Ligand 8 with the wild type receptor showed no key hydrogen bond interactions. The presence of hydrophobic and electrostatic interactions could contribute to the better binding affinity value of −7.7 kcal/mol (Figures 6 and 7).

Earlier *in vitro* and *in vivo* studies have shown an increased activity of moxifloxacin-conjugated dansylated carboxymethylglucan (M-DCMG) than free moxifloxacin against persisting *M*. *tuberculosis* within macrophages [36]. But this conjugation is not supportive for conjugation with PA-824 since Carboxymethylglucan has been shown to have antioxidant activities [37] which could counteract the essential ROS generation by PA-824 for bactericidal activity. Interestingly, it is also pointed that the efficacy of M-DCMG in improving the activity of Moxifloxacin [36] was that its ability to localize with the persisting tissues of C57BL/6 mice infected with *M*. *tuberculosis*. Since PA-824 is known to localize to persisting tissues [19], its conjugation with moxifloxacin could provide a better therapeutic advantage against the persistors. Wang *et al.,*[38] noted that fuoroquinolones such as moxifloxacin, appear to show enhanced action in the presence of ROS. This support the enhanced structure-activity relationship against *M*. *tuberculosis* by ligand 8, which is a combinatorial structure of moxifloxacin with an ROS/RNS generator - PA-824. The support for the ester linkage for the structure activity of the combinatorial drug design is provided by the work by Georgopapadakou and Bertasso, [39], who showed that cephalosporin 3′-quinolone ester (Ro 23–9424) is effective in cases when neither of its individual components, cephalosporin and quinolone, are active. In the same way when there is resistance for moxifloxacin and PA-824 as individual drugs, the ester combination of both (ligand 8) could have a synergistic activity against *M*. *tuberculosis* which could help in combination therapy. Further, since ligand 8 showed binding at the hydrophobic pocket (red colour) of the Ddn receptor (Figure 6), it can be considered that the ligand has more of hydrophobic interactions. This feature could maintain the stability of the ester bond in the presence of plasma and esterases as described by Simões *et al*., [40]. A combination treatment of rifampin, moxifloxacin, amikacin and PA-824 has shown potent killing of MTB *in vitro* in 14 days [41]. Recently, another study of phase II clinical trials in South Africa, the combination therapy PaMZ, which consists of the novel TB drug candidate PA-824, moxifloxacin and pyrazinamide killed more than 99 per cent of people’s TB bacteria within just 14 days and is potentially suitable for treating both drug sensitive and multidrug resistant tuberculosis [42]. Thus evaluation of treatment strategies applying combinatorial/synergistic activities could have a positive impact in the treatment of TB.

## Conclusions

In summary PA-824 exhibited greater bactericidal activity on non-replicating organisms (persisters) under normal pH than that of RIF and PZA, which may help in shortening the duration of treatment. Interestingly, the dose of 12.5 μg/ml and 21 days treatment was observed to have an ability to reduce the bacterial count to zero, which may offer key insights while setting the doses for *in vivo*/clinical studies. From the combinatorial analysis, ligand 8 (PA-824-Moxifloxacin ester conjugate) showed the most potent activity against both wild type and mutant Ddn receptors and hence needs further *in vitro* investigation of its enantiomeric binding properties with the Ddn receptor.

## Competing interests

The authors declare that they have no competing of interests.

## Authors’ contributions

CNP, SS have designed the work. SS and RSA carried out the experiment. PV analyzed the data and contributed for the statistical analysis. SS and RSA wrote the manuscript and CNP reviewed the manuscript critically. All the authors have read the article and approved the final manuscript.
